# Molecular detection and characterization of malignant catarrhal fever viruses in Namibia: insights into OvHV-2 and AlHV-1 presence at the wildlife–livestock interface

**DOI:** 10.1007/s11259-026-11560-2

**Published:** 2026-10-08

**Authors:** Umberto Molini, Lauren M. Coetzee, Ellini Hamunyela, Juliet Kabajani, William G. Dundon, Giovanni Franzo

**Affiliations:** 1 Central Veterinary Laboratory (CVL), 24 Goethe Street, 18137 Windhoek, Namibia; 2 Istituto Zooprofilattico Sperimentale dell’Abruzzo e del Molise, 64100 Teramo, Italy; 3 Animal Production and Health Laboratory, Animal Production and Health Section, Joint FAO/IAEA Centre of Nuclear Techniques in Food and Agriculture, Department of Nuclear Sciences and Applications, International Atomic Energy Agency, Wagramer Strasse 5, P.O. Box 100, A1400 Vienna, Austria; 4 Department of Animal Medicine, Production and Health (MAPS), University of Padova, Legnaro, Italy

**Keywords:** Malignant catarrhal fever, Namibia, OvHV-2, AlHV-1, Molecular epidemiology

## Abstract

Malignant catarrhal fever (MCF) is a severe lymphoproliferative disease of domestic and wild ruminants caused by macaviruses, mainly ovine gammaherpesvirus 2 (OvHV-2) and alcelaphine gammaherpesvirus 1 (AlHV-1). Although relevant in sub-Saharan Africa, molecular epidemiological data remain limited, particularly in Namibia. This study investigated the occurrence and diversity of MCF viruses in Namibian livestock and contextualised local strains globally.

Fifteen samples from cattle and sheep suspected of MCF were collected across five Namibian regions in 2023. Viral DNA was screened by duplex nested PCR, followed by amplification and sequencing of selected OvHV-2 (ORF50, ORF75, Ov9.5) and AlHV-1 (ORF50, A9.5) genomic regions. Phylogenetic and genetic distance analyses assessed viral diversity.

OvHV-2 was the most commonly reported, being detected in 13/15 samples from both symptomatic cattle and asymptomatic sheep, whereas AlHV-1 was identified in two bovine cases. OvHV-2 sequences showed limited diversity and clustered into two main clades, without clear geographic structuring and with intermixing between host species. Several Namibian strains were closely related to sequences from Africa, Europe, Asia, and North America, depending on the genomic region analysed. AlHV-1 sequences were related to strains from southern and eastern Africa.

These findings support widespread OvHV-2 circulation and indicate sheep as a likely infection source for cattle. The lack of spatial structure and presence of multiple clades suggest complex transmission dynamics, potentially influenced by animal movements, although the role of livestock trade and wildlife-mediated transmission remains unclear. Enhanced molecular surveillance, biosecurity, and control of animal translocations are needed to mitigate MCF impact in the region.

## Introduction

Malignant catarrhal fever (MCF) is a frequently fatal, lymphotropic and vasotropic viral disease caused by several agents belonging to the malignant catarrhal fever virus (MCFV) complex (Russell et al. 2009), within the subfamily *Gammaherpesvirinae*, genus *Macavirus*, currently including 9 viral species *(ICTV* 2026; *accessed 13/04/2026)*. These viruses infect a wide range of hosts within the order *Artiodactyla* (cloven-hoofed animals), including species from the families *Bovidae*, *Giraffidae*, *Cervidae*, and *Suidae* (Li et al. 2014). Infections have also been reported in *Equidae*, belonging to the order *Perissodactyla* (odd-toed ungulates)(Costa et al. 2009).

*Alcelaphine gammaherpesvirus 1* (AlHV-1) and *Ovine gammaherpesvirus 2* (OvHV-2) are the most extensively studied *Macavirus* species and are considered of major economic relevance, particularly in certain regions, due to the high mortality observed in susceptible species *(*Pierson et al. 1973; O’Toole et al. 1997; Li et al.^2006^; Swai et al. 2013; Cleaveland et al. 2001). These viruses are responsible for the two main epidemiological forms of the disease: sheep-associated MCF (SA-MCF) and wildebeest-associated MCF (WA-MCF), also known as the African form.

The viruses are characterized by a monopartite, linear, dsDNA genome of approximately 130 kbp, bounded by terminal repeats of 1.1 kbp for AlHV-1 and 4.2 kbp for OvHV-2 At least 70 open reading frames (ORFs) have been identified in AlHV-1, and 73 in OvHV-2 (Ensser et al. 1997; Hart et al. 2007).

AlHV-1 has two species of wildebeest as its reservoir: the blue wildebeest (brindled gnu, *Connochaetes taurinus*) and the black wildebeest (white-tailed gnu, *C. gnou*) (Gong et al. 2023; Li et al. 2014; Plowright et al. 1960). The blue wildebeest is widely distributed across sub-Saharan Africa, whereas the black wildebeest has a much more limited range in southern Africa and is now largely confined to protected reserves and game parks. In free-ranging populations, the prevalence of infection is nearly 100%, and infected wildebeest remain completely asymptomatic. However, several WA-MCF-susceptible species, including cattle, bison, deer, buffalo, and other related antelopes, have been identified (Li et al. 2014; Gong et al. 2023).

In contrast, SA-MCF has a global distribution and is caused by OvHV-2, with sheep as the reservoir, with sporadic outbreaks reported in cattle, bison, deer, pigs and giraffe. (Li et al. 2014)

From an epidemiological perspective, both vertical and horizontal transmission occur in wildebeest. Young wildebeest appear to become infected before the fourth month of life, mainly via the nasal route from animals excreting free viral particles, although the proportion of vertical (transplacental) transmission remains unclear. Infected wildebeest calves develop viraemia and shed virus through ocular and nasal secretions. In sheep the main route is aerosol transmission from adolescent animals (6–9 months) that shed large amounts of virus (Mushi et al. 1981). Transplacental and milk transmission are rare and, overall, most lambs are not infected with OvHV-2 until after two months of age (Li et al. 1998). Nasal shedding is the primary route of OvHV-2 transmission (Li et al. 2004), although other secretions may be involved (Hüssy et al. 2002).

Clinically susceptible species acquire infection through inhalation or ingestion of virus-containing secretions, or possibly through contaminated feed or water. Long-distance transmission of OvHV-2 from sheep to bison has been documented (Li et al. 2008). The role of mechanical vectors, such as birds, in long-distance transmission remains unclear.

AlHV-1 and OvHV-2 are not naturally transmitted between clinically susceptible hosts and therefore, animals affected by MCF do not represent a significant risk for transmission to other susceptible animals (Berezowski et al. 2005; Li et al. 2014) .

When transmitted to susceptible species, AlHV-1 and OvHV-2 cause an indistinguishable acute systemic condition characterised by lymphoproliferation and widespread inflammation, particularly affecting mucosal tissues and blood vessels (Li et al. 2014; O’Toole and Li 2014).

The incubation period is highly variable, ranging from weeks to months, and may be influenced by several factors, including the infectious dose, which has been positively correlated with disease onset. Clinical signs typically begin with depression and fever (40–42 °C), followed by oculonasal discharge. Disease progression varies among species depending on their susceptibility (Russell et al. 2009).

In cattle, the most common presentation is the so-called head-and-eye form, characterised by bilateral nasal discharge that becomes mucopurulent, profuse, and occasionally haemorrhagic. Affected animals may exhibit dyspnoea and open-mouth breathing. Severe bilateral keratitis, with corneal opacity, excessive lacrimation, and accumulation of pus in the anterior chamber, is frequently observed, leading to photophobia and impaired vision. Diarrhoea and neurological signs, such as circling, may also occur (O’Toole and Li 2014). In highly susceptible species, such as American bison and many cervids, the disease course is typically shorter than in cattle and may result in sudden death (Headley et al. 2020; Gong et al. 2023).

Although OvHV-2 infection in sheep is typically subclinical and viral DNA can commonly be detected by PCR in clinically healthy animals, MCF-like disease has occasionally been reported in sheep. In these cases, the association with OvHV-2 has been supported by compatible histopathological lesions combined with molecular detection of the virus, including PCR and in situ hybridization. In these cases, the viral load was found to be 100–1000 times higher than in asymptomatic animals. This suggests that, under certain conditions, sheep may also develop histopathological lesions consistent with MCF (Phillips et al. 2018; Pesavento et al. 2019; Sheehan et al. 2024) .

In Africa, the disease has been documented in several countries, including Tanzania, Ethiopia, Kenya, South Africa, Zambia, Zimbabwe, Mozambique, Namibia, Botswana, and Angola (Gelaye et al. 2013; Wambua et al. 2016; Shiba et al. 2026).

Cattle farmers experience significant losses each year due to wildebeest-transmitted MCF (Swai et al. 2013; Mlilo et al. 2015; Gong et al. 2023). The incidence of MCF is higher on commercial farms, where cattle and wildebeest tend to graze together and wildebeest populations are more abundant. A study performed in Zimbabwe reported MCF accounted for 71% and 21% of cattle mortalities in the commercial and smallholder sectors, respectively (Mlilo et al. 2015). However, for smallholder and communal farmers, cattle losses due to MCF extend beyond immediate production impacts, undermining asset accumulation, herd growth, and overall household resilience. Therefore, reduction or elimination of the disease would allow for a significant increase in pastoral livelihood profitability (Lankester et al. 2015b).

Despite the relevance of the topic, data on MCF presence, typing and epidemiology, particularly molecular epidemiology are scarce in the African country. The present study aimed to address these gaps by testing and genetically characterising AlHV-1 and OvHV-2 samples collected from different Namibian districts and contextualising them within the international scenario.

## Materials and methods

### Sample collection and processing

**Fig. 1:**
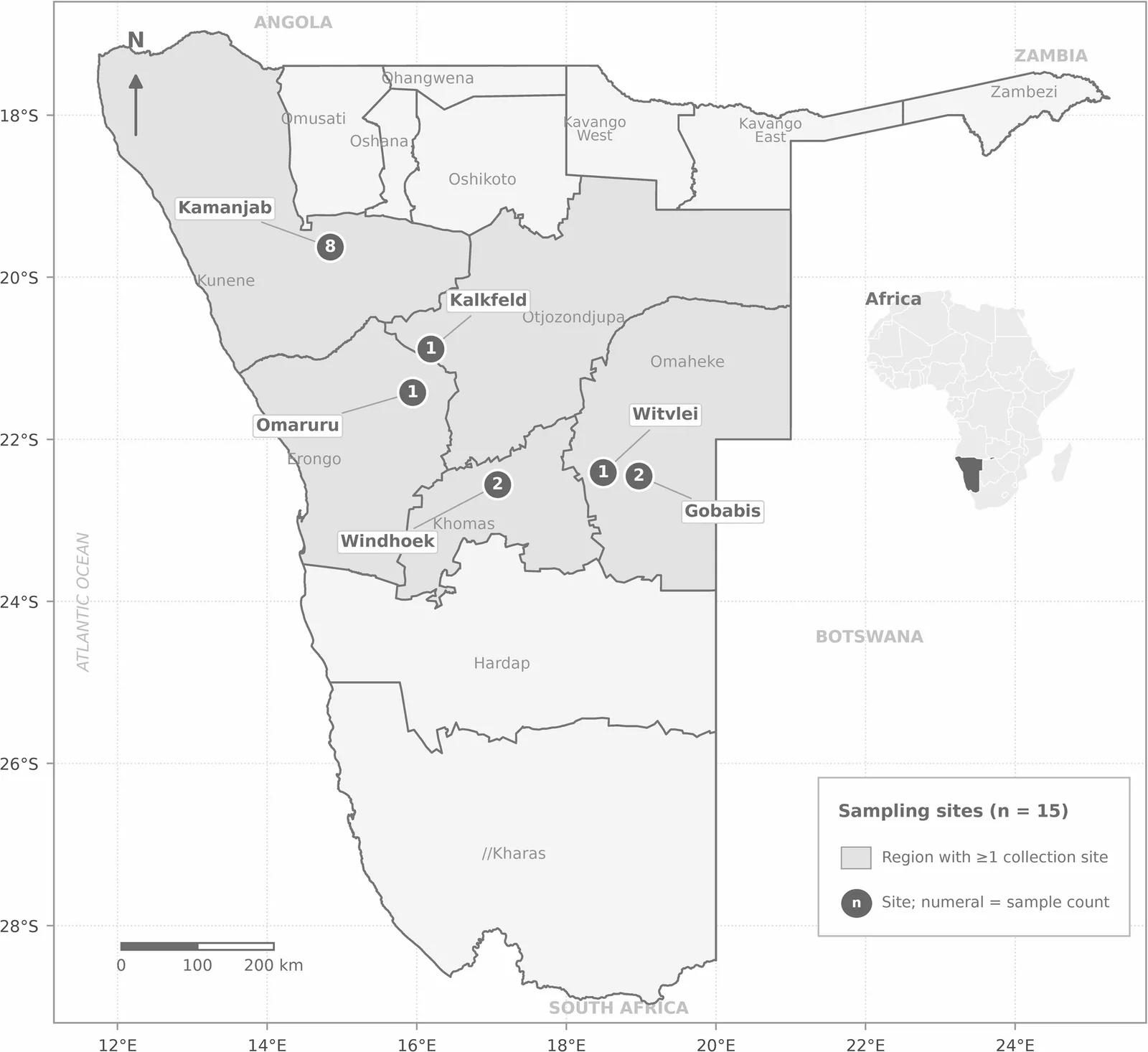
Geographic distribution of sampling sites included in the study in Namibia. The six sampling localities comprised Kamanjab (n = 8), Kalkfeld (n = 1), Omaruru (n = 1), Windhoek (n = 2), Witvlei (n = 1), and Gobabis (n = 2), for a total of 15 samples. The map was generated in Python using Matplotlib version 3.10.8. First-level administrative boundaries were obtained from the Namibia Statistics Agency dataset distributed through geoBoundaries (Runfola et al., 2020), and locality coordinates were sourced from GeoNames.Sampling localities were displayed as uniformly sized circles, with numerals indicating the number of samples from each locality; administrative regions containing sampling localities were shaded. Points represent the geographical positions of the named localities rather than precise farm coordinates

The study included EDTA-anticoagulated blood samples from 15 ruminants (bovine and ovine) suspected of malignant catarrhal fever (MCF). Samples were collected by state veterinarians between May and September 2023 from communal rural farms located in five regions of Namibia (Kunene, Otjozondjupa, Omaheke, Khomas, and Erongo) (Fig. 1; Table 1) and submitted to the Central Veterinary Laboratory (Windhoek, Namibia) for diagnostic confirmation. All sheep were clinically asymptomatic but housed in farms were affected cattle were present. In contrast, cattle exhibited bilateral ocular and nasal discharge, dry muzzle, fever, and lateral recumbency, frequently accompanied by mucopurulent reddish nasal discharge and crusting of the eyes and nose pad. Bilateral corneal opacity (“blue eye”) was detected in two animals.

**Table 1:** Summary of samples included in the study of malignant catarrhal fever viruses (MCFVs) in Namibia. Corresponding sequence accession numbers are provided for each sequenced gene

ID	Location	Region	Specie	Age	Sex	Year	Typing	OvHV-2	OvHV-2	OvHV-2	AlHV-1 ORF50	AlHV-1 A9.5
								ORF75	ORF50	OV 9.5		
5593	Kamanjab	Kunene	bovine	1y	M	2023	OvHV-2	PZ193153	PZ193138	PZ193164		
6058	Kalkfield	Otjozondjupa	bovine	2y	F	2023	AlHV-1				PZ193140	PZ221388
6118-1	Kamanjab	Kunene	ovine	1y	F	2023	OvHV-2		PZ193137	PZ193163		
6118-2	Kamanjab	Kunene	ovine	1y	F	2023	OvHV-2	PZ193148	PZ193136	PZ193165		
6118-3	Windhoek	Khomas	ovine	1y	M	2023	OvHV-2	PZ193147	PZ193135	PZ193162		
6118-4	Windhoek	Khomas	ovine	1y	F	2023	OvHV-2	PZ193146	PZ193134	PZ193161		
6118-5	Gobabis	Omaheke	ovine	1y	F	2023	OvHV-2	PZ193150	PZ193133	PZ193160		
6120-2	Witvlei	Omaheke	ovine	1y	F	2023	OvHV-2	PZ193145	PZ193132	PZ193159		
6120-3	Kamanjab	Kunene	ovine	1y	F	2023	OvHV-2	PZ193144	PZ193131	PZ193158		
6120-5	Kamanjab	Kunene	ovine	1y	M	2023	OvHV-2	PZ193143	PZ193130	PZ221390		
6121-2	Kamanjab	Kunene	ovine	1y	F	2023	OvHV-2	PZ193142	PZ193129	PZ193157		
6245	Omaruru	Erongo	bovine	4y	F	2023	AlHV-1				PZ193139	PZ221387
7286	Gobabis	Omaheke	bovine	1y	F	2023	OvHV-2	PZ193152	PZ193128	PZ193156		
7287-2	Kamanjab	Kunene	ovine	1y	F	2023	OvHV-2	PZ193151	PZ193127	PZ193155		
7287-3	Kamanjab	Kunene	bovine	1y	F	2023	OvHV-2	PZ193141	PZ193126	PZ193154		

Total genomic DNA was extracted from 200 µL of whole blood using the High Pure Viral Nucleic Acid Kit (Hoffmann-La Roche, Switzerland), with a final elution volume of 100 µL, according to the manufacturer’s instructions. DNA extracts were screened using a single-tube duplex nested PCR assay (Bremer et al. 2005) for the simultaneous detection and differentiation of ovine gammaherpesvirus 2 (OvHV-2) and alcelaphine gammaherpesvirus 1 (AlHV-1).

For OvHV-2–positive samples, ORF50, ORF75, and Ov9.5 gene fragments were amplified by nested PCR as previously described (Russell et al. 2014).

For AlHV-1–positive samples, ORF50 and A9.5 gene fragments were amplified. The A9.5 gene was amplified by nested PCR as previously described (Russell et al. 2013) while the AlHV-1 ORF50 fragment was amplified in a single PCR using primers AHVorf50_F and AHVorf50_R (Lankester et al. 2015a).

Amplicons from positive samples were purified using the Wizard SV Gel and PCR Clean-Up System (Promega, USA) and commercially sequenced by LGC Genomics (Berlin, Germany). Sequence data were edited and assembled using the Staden software package v2.0.0b8 and submitted to the GenBank database (Table 1).

### Phylogenetic analysis 

For each genomic region analysed (OvHV-2 ORF75, OvHV-2 ORF9.5, OvHV-2 ORF50, AlHV-1 A9.5, and AlHV-1 ORF50), homologous sequences were identified by searching in GenBank. Candidate sequences were selected based on their overlap with the genomic regions generated in this study, ensuring sufficient coverage of the target loci.

Selected sequences were downloaded and, when available, annotated with associated metadata, including the sampling date, host species, and country of origin. The resulting datasets were curated separately for each genomic region.

Multiple sequence alignments were performed using MAFFT (Katoh and Standley 2013) with default parameters. Alignments were subsequently inspected and trimmed to the appropriate length to match the sequenced regions and to remove poorly aligned or non-overlapping positions.

Phylogenetic relationships were inferred using the maximum likelihood method implemented in IQ-TREE (Trifinopoulos et al. 2016). For each dataset, the best-fitting nucleotide substitution model was automatically selected by IQ-TREE according to the Bayesian Information Criterion (BIC). Branch support was assessed using 10,000 ultrafast bootstrap replicates.

## Results

### Sequencing results

A total of 15 samples collected in 2023 from different Namibian districts were analysed, of which 5 originated from cattle and 10 from sheep, collected across five Namibian regions: Kunene, Otjozondjupa, Khomas, Omaheke, and Erongo. Sequences were successfully obtained for multiple genomic regions of OvHV-2 (ORF75, ORF50, and Ov9.5) and AlHV-1 (ORF50 and A9.5) (Table 1). OvHV-2 was the most frequently detected virus, identified in 13 out of 15 samples .Most OvHV-2-positive samples yielded complete data for all three targets, with only occasional partial amplification (i.e. sample 6118-1) for which ORF75 could not be amplified). In contrast, AlHV-1 was detected in two samples and two genomic regions (ORF50 and A9.5) were obtained for each sample.

From a geographical perspective, OvHV-2 showed a wide distribution across Namibia, being detected in multiple regions, including Kunene, Khomas, and Omaheke, and in both ovine and bovine hosts. Conversely, AlHV-1 displayed a more restricted spatial distribution, being identified only in the Otjozondjupa and Erongo regions and exclusively in cattle.

### Genetic and phylogenetic analysis of ovine gammaherpesvirus 2

Analyses were performed on different datasets due to the limited overlap in sequencing among genomic regions, in order to maximise the number of comparable sequences. In the Ov9.5 region, the p-distance (i.e. the proportion of mismatched positions between two DNA sequences) among Namibian sequences was mean = 0.165 (min-max = 0- 0.316), while the overall dataset showed the following distances: mean = 0.245 (min-max = 0- 0.316).

**Fig. 2:**
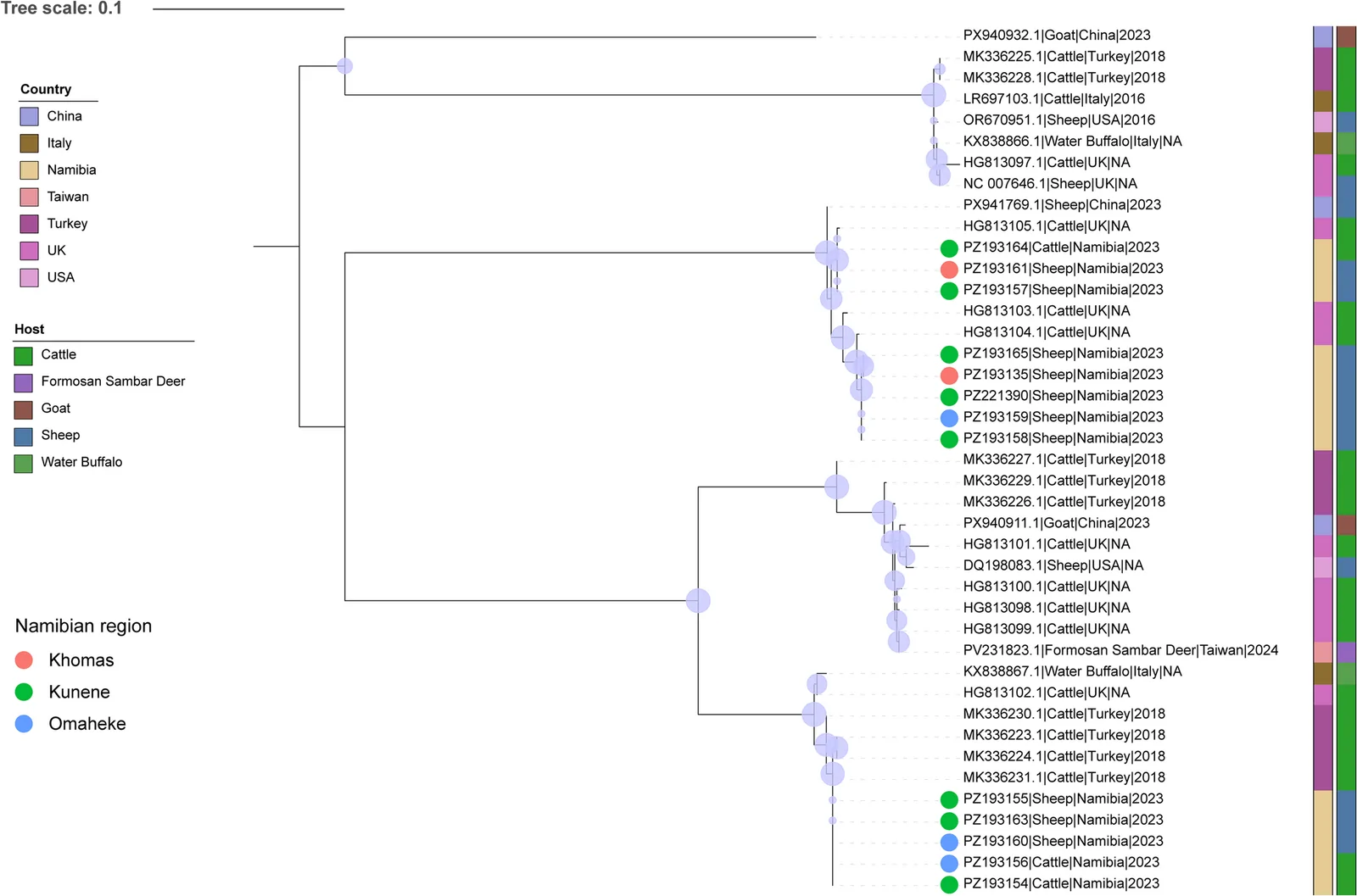
Phylogenetic analysis of the Ov9.5 gene of ovine gammaherpesvirus 2 (OvHV-2). The analysis included 41 sequences aligned over 1,019 nucleotide positions. The tree was inferred using the TPM3u+F+G4 nucleotide substitution model, selected as the best-fit model according to the Bayesian Information Criterion (BIC). Tip labels report the GenBank accession number, host species, country of origin, and collection date or year, where available. Sequences generated in the present study are indicated by coloured circles, with colours representing the Namibian region of origin (Khomas, Kunene, or Omaheke). Node support is represented by shaded circles, with circle size proportional to the support value. Host species and country of origin are additionally indicated by colour-coded strips adjacent to the corresponding tip labels. The scale bar indicates the number of nucleotide substitutions per site

Phylogenetically, Namibian sequences clustered into two main clades (Fig. 2). One included sequences from the United Kingdom and China, whereas the other showed a closer relationship with Turkish strains, although Italian and UK sequences were also present within the same clade. Notably, sequences obtained from Namibian bovine samples were distributed across both clades, and overall, strains from different host species were intermixed within the phylogenetic tree.

Regarding the geographical distribution within Namibia, no clear spatial clustering was observed. Despite the limitations imposed by the sample size, sequences collected from different regions were often identical or showed high genetic similarity and were intermixed in the phylogenetic tree, suggesting a lack of evident geographic structuring.

In the ORF50 region, the p-distance among Namibian sequences was mean = 0.005 (min-max = 0- 0.009), while the overall dataset showed the following distances: mean = 0.007 (min-max = 0- 0.0187).

**Fig. 3:**
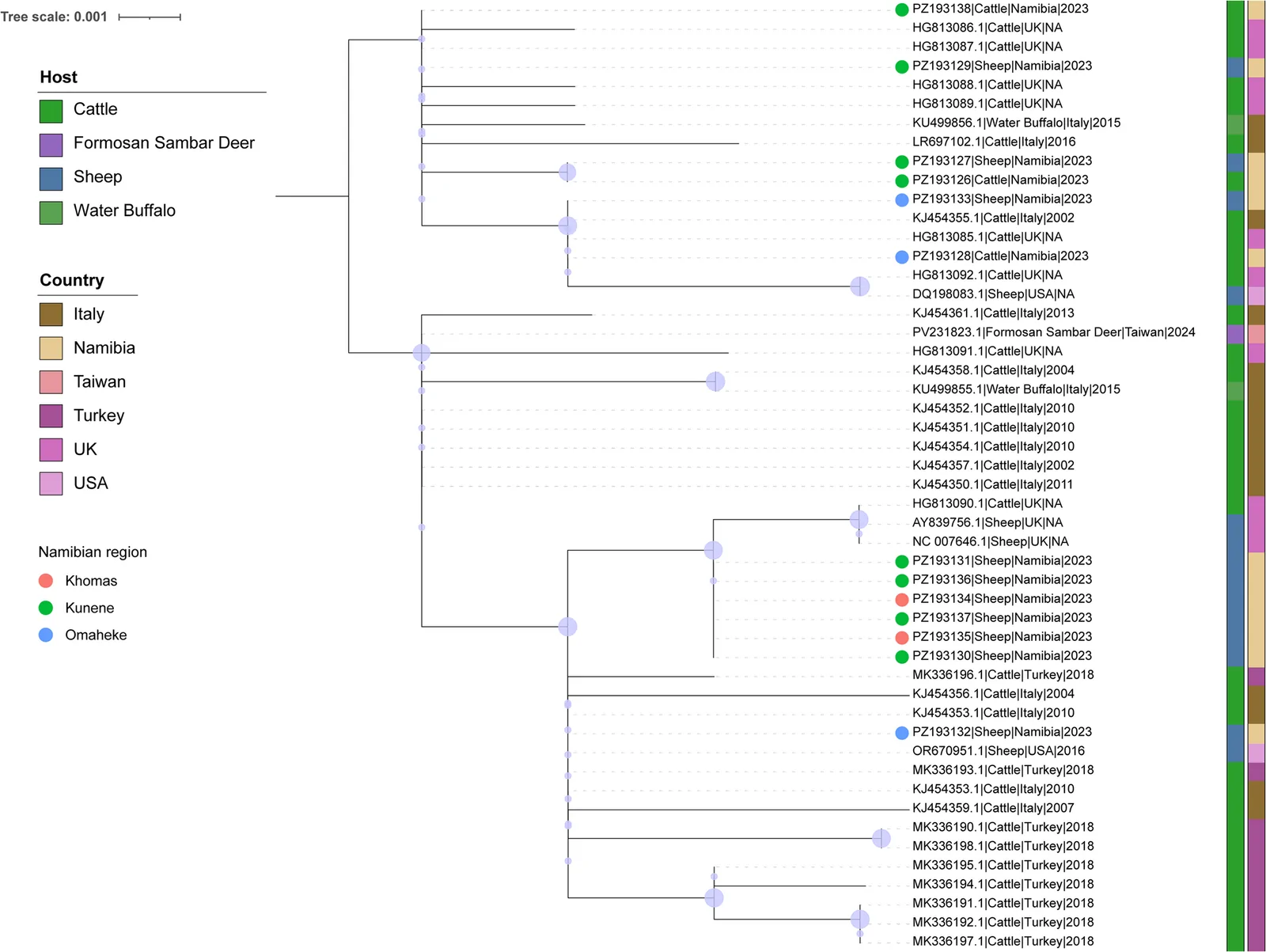
Phylogenetic analysis of the ORF50 gene of ovine gammaherpesvirus 2 (OvHV-2). The analysis included 50 sequences aligned over 420 nucleotide positions. The tree was inferred using the HKY+F nucleotide substitution model, selected as the best-fit model according to the Bayesian Information Criterion (BIC). Tip labels report the GenBank accession number, host species, country of origin, and collection date or year, where available. Sequences generated in the present study are indicated by coloured circles, with colours representing the Namibian region of origin (Khomas, Kunene, or Omaheke). Node support is represented by shaded circles, with circle size proportional to the support value. Host species and country of origin are additionally indicated by colour-coded strips adjacent to the corresponding tip labels. The scale bar indicates the number of nucleotide substitutions per site

Also in this case Namibian sequences clustered into two main clades (Fig. 3) with heterogenous geographical composition. One clade included sequences from Italy, Turkey, UK and US, whereas the other showed a closer relationship with Italy and the UK. Bovine samples were distributed across both clades, and overall, strains from different host species were intermixed within the phylogenetic tree. No clear regional clustering was observed in Namibia.

**Fig. 4:**
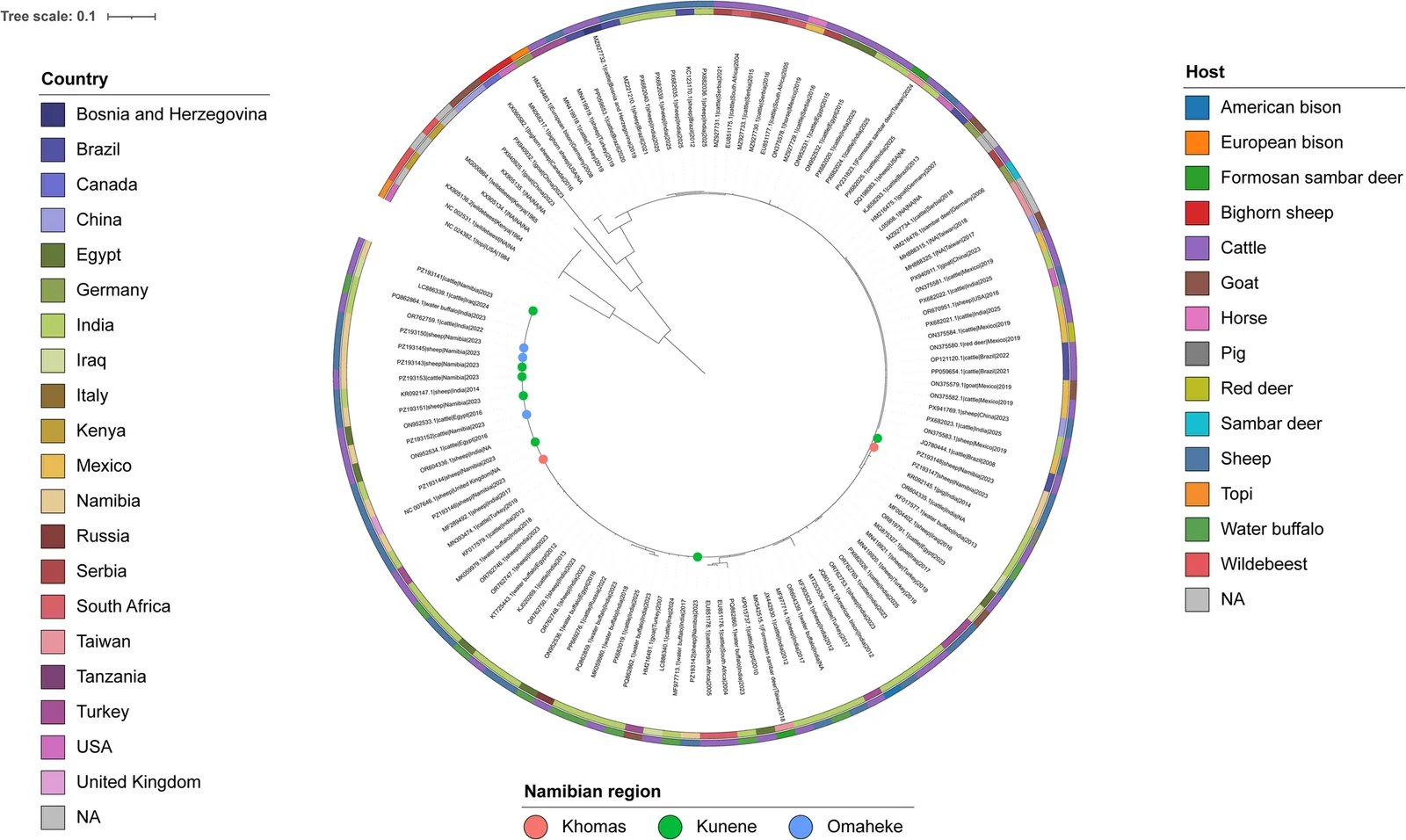
Phylogenetic analysis of the ORF75 gene of ovine gammaherpesvirus 2 (OvHV-2). The analysis included 118 sequences aligned over 396 nucleotide positions. The tree was inferred using the K2P+G4 nucleotide substitution model, selected as the best-fit model according to the Bayesian Information Criterion (BIC). Tip labels report the GenBank accession number, host species, country of origin, and collection date or year, where available. Sequences generated in the present study are indicated by coloured circles, with colours representing the Namibian region of origin (Khomas, Kunene, or Omaheke). Node support is represented by shaded circles, with circle size proportional to the support value. Host species and country of origin are additionally indicated by colour-coded strips adjacent to the corresponding tip labels. The scale bar indicates the number of nucleotide substitutions per site. A more detailed visualization of the same tree is reported as Supplementary figure 1

ORF75 dataset benefitted from a higher reference sequence number, although the low genetic variability limited analysis resolutions (Fig. 4). Namibian sequences showed a mean p-distance of 0.0007 (min-max = 0-0.002) while the variability in the overall dataset was: mean 0.006 (min-max = 0-0.043).

### Genetic and phylogenetic analysis of alcelaphine gammaherpesvirus 1

**Fig. 5:**
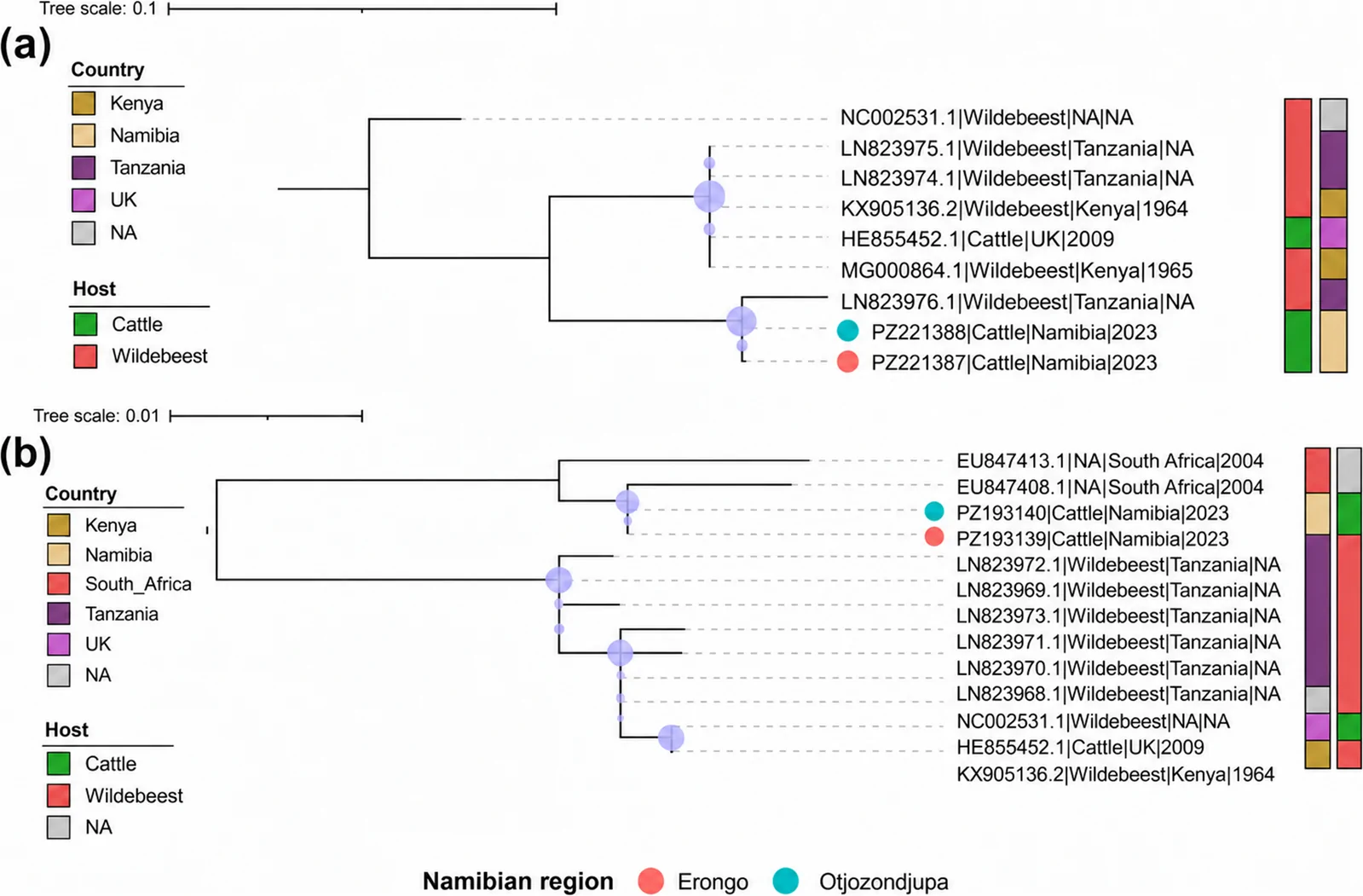
Phylogenetic analysis of alcelaphine herpesvirus 1 (AlHV-1). Maximum Likelihood phylogenetic trees were reconstructed based on the (**a**) A9.5 gene and (**b**) ORF50. For A9.5 gene the analysis included 9 sequences aligned over 699 nucleotide positions. The tree was inferred using the TPM2u+F nucleotide substitution model, selected as the best-fit model according to the Bayesian Information Criterion (BIC) while for ORF50 the analysis included 13 sequences aligned over 516 nucleotide positions. The tree was inferred using the K2P nucleotide substitution model, selected as the best-fit model according to the Bayesian Information Criterion (BIC). Tip labels report the GenBank accession number, host species, country of origin, and collection date or year, where available. Sequences generated in the present study are indicated by coloured circles, with colours representing the Namibian region of origin (Erongo or Otjozondjupa). Node support is represented by shaded circles, with circle size proportional to the support value. Host species and country of origin are additionally indicated by colour-coded strips adjacent to the corresponding tip labels. The scale bar indicates the number of nucleotide substitutions per site

In the A9.5 region, the p-distance between the two Namibian sequences was 0.003, and they showed the closest relationship to a strain collected from a blue wildebeest in Tanzania. (Fig. 5a). On the other hand, in the ORF50 the 2 identical Namibian sequences were part of a clade including South African strains (Fig. 5b).

## Discussion

Livestock production remains a cornerstone of rural livelihoods in sub-Saharan Africa, providing income, food security, and economic stability for millions of households (Erdaw 2023). However, these resources are continuously threatened by animal infectious diseases, whose dynamics are increasingly shaped by environmental changes, land-use modifications, and interactions at the wildlife–livestock interface, particularly in areas where communal grazing overlaps with protected areas and private game farms (Shiba et al. 2026).

Malignant catarrhal fever (MCF), although of relatively limited relevance in Western countries, represents a major disease affecting domestic ruminants in Africa. The wildebeest-associated form has a significant impact in sub-Saharan regions, where it can account for up to 10% of annual cattle losses (Orono et al. 2019). In addition to direct economic losses, strategies aimed at avoiding contact with wildebeest often involve moving cattle to lower-quality pastures, resulting in reduced productivity and an increased prevalence of other diseases, thereby further compromising herd profitability (Gong et al. 2023).

These effects are closely linked to husbandry practices and the degree of contact between reservoir and susceptible hosts. In communal farming systems, the co-presence of sheep and cattle may favour OvHV-2 exposure, while the local presence and movements of wildebeest may increase the risk of AlHV-1 transmission, particularly during the calving period (Li et al. 1998, 2004; Mushi et al. 1981; Wambua et al. 2016). However, detailed information on wildebeest occurrence, movements and contact with livestock was unavailable for the sampled farms.

Information on the epidemiology of MCF in Africa remains limited and is largely based on clinical and serological evidence or on diagnostic approaches that do not allow proper strain characterisation. The present study describes, for the first time, the molecular detection and genetic characterisation of MCFV strains associated with suspected clinical cases in Namibia. Despite the hypothesised pivotal role of AlHV-1 in the African context, most of the detected strains were identified as OvHV-2, including those obtained from cattle showing clinical signs. OvHV-2 DNA was detected in clinically suspected bovine MCF cases, supporting its likely involvement, although confirmatory pathological data were not available. Therefore, the epidemiological role of OvHV-2 in this setting warrants further investigation.

A close genetic relationship was observed between strains circulating in Namibian sheep and cattle, supporting the role of sheep as a likely source of infection for susceptible species. Additionally, the absence of significant viral variability in these two species might indicate absence of evolution in the epidemiological dead-end host. At the same time, the detection of OvHV-2 across multiple regions indicates widespread circulation within the country, which is likely associated to the animal movements in Namibia, sheep particularly. The more variable genomic regions, such as Ov9.5 and ORF50, revealed the presence of at least two major genetic clusters co-circulating in Namibia, with limited intra-cluster variability. However, no clear geographic structuring was observed, as strains belonging to different clades were detected within the same region and, conversely, individual regions harboured multiple OvHV-2 lineages.

Such a pattern is consistent with the subclinical nature of OvHV-2 infection in the reservoir host, which reduces the likelihood of targeted diagnostic investigations and may facilitate undetected viral circulation. At the same time, the occurrence of OvHV-2-positive cattle showing clinical signs compatible with MCF in different regions and across distinct genetic clusters highlights the importance of implementing effective control strategies to limit viral transmission and mitigate its impact on animal health and productivity.

The co-circulation of distinct clades in Namibia is consistent with the presence of multiple viral lineages within the country. The observed clustering patterns are compatible with the circulation of multiple viral lineages in Namibia; however, the limited number of comparable sequences and the use of short genomic fragments do not allow robust inference on the geographic origin or number of introduction events. The clustering of Namibian strains with sequences from Asia, Europe, and North America likely reflects the widespread global distribution of OvHV-2, although this interpretation is constrained by the disproportionate availability of sequences, particularly from African countries. Additionally, the importation of sheep from non-African countries is minimal, largely due to the susceptibility of non-local breeds to diseases such as bluetongue, which is likely associated to sheep movement in Namibia (Zulu & Venter 2014; Ojango et al. 2023). Thus, alternative sources of introduction should be considered.

Similarly, the identification of genetic similarity between Namibian strains and those reported from other regions, including the Middle East, may reflect broad patterns of viral circulation rather than direct epidemiological links. The limited availability of well-annotated reference sequences, especially from Africa, precludes robust inference on transmission pathways and sources of introduction. This limitation, also highlighted in previous studies (Bianchessi et al. 2022), represents a major constraint to understanding the epidemiology of MCF in this context.

Molecular data on AlHV-1 are even more limited. In this study, the detected sequences were almost identical, which might testify a common source of infection, although the limited variability in the considered region might also be involved. Those strains showed genetic similarity with strains reported from eastern and southern Africa, which may be consistent with regional circulation patterns and the involvement of wildebeest populations. However, the relationship observed with sequences from Tanzania, despite a certain degree of genetic similarity, is unlikely to reflect direct natural dispersal mediated by wildebeest movements. Wildebeest migrations are typically regional (approximately 200–300 km), with the well-known long-distance movements in the Serengeti ecosystem only representing a specific ecological context. Given the considerable geographic distance and environmental differences between Tanzania and Namibia, direct contact through wildlife movements appears unlikely.

In addition, the farms included in this study are located within areas of Namibia officially recognised as free from foot-and-mouth disease (FMD), where strict biosecurity measures are in place. These include veterinary cordon fences designed to limit the movement of wild animals and reduce contact between wildlife and domestic livestock. Furthermore, commercial farming systems are typically enclosed by game-proof fencing, which further restricts interactions with external ruminant populations. Nevertheless, the voluntary introduction of wildebeest into farms for game-related purposes, often without prior testing, represents a potential risk factor for MCF outbreaks. The detection of identical strains in cattle from neighbouring regions may be more plausibly explained by livestock movement or trade, although alternative transmission pathways cannot be excluded.

Therefore, considering the wildlife origin of the infection, the significant impact on domestic livestock, and the relative feasibility of implementing protective measures at the farm level, the introduction of non-native animals should be carefully regulated. Alternatively, appropriate diagnostic screening and quarantine measures should be established and strictly enforced.

Despite the insights provided, this study presents several limitations that should be acknowledged. At first, the limited sample size dictate caution in the interpretation of the results, particularly concerning the inference of epidemiological links among Namibian samples. At a broader scale, the number of available sequences, particularly from African countries, remains limited, and the uneven availability of genomic regions across datasets has constrained comparative analyses. In addition, the reliance on partial genomic regions, although informative, may have limited the resolution of phylogenetic and phylogeographic inferences. The lack of comprehensive and standardised metadata (e.g. precise geographic origin, host information and sampling context) further limited the ability to reconstruct transmission pathways and to robustly infer epidemiological links.

Future studies should aim to expand both the number and representativeness of sampled strains, with particular emphasis on underrepresented regions and hosts. The adoption of whole-genome sequencing approaches, coupled with harmonised sampling strategies and standardised data reporting, will be essential to improve the resolution of molecular epidemiological analyses. In parallel, integrating molecular data with detailed epidemiological and ecological information, including animal movement patterns and wildlife–livestock interactions, will be crucial to better understand transmission dynamics.

Ultimately, such efforts will not only enhance our understanding of MCF epidemiology in Africa but also support the development of more effective surveillance, prevention and control strategies, particularly in settings where wildlife–livestock interfaces play a central role.

## Data Availability

All obtained sequences have been made freely available in GenBank.
